# Designing the structure of cationic star-shaped trimeric surfactants most active in micelle formation using molecular connectivity indices

**DOI:** 10.1038/s41598-024-58854-6

**Published:** 2024-04-09

**Authors:** Anna Mozrzymas

**Affiliations:** Department of Physics and Biophysics, Wrocław University of Environmental and Life Sciences, ul. Norwida 25, 50-375 Wrocław, Poland

**Keywords:** Polymer chemistry, Chemistry, Computational chemistry

## Abstract

A model expressing the relationship between the logarithm of critical micelle concentration of cationic star-shaped trimeric surfactants and topological indices was obtained using only molecular connectivity indices. Based on the obtained model, the exemplary compound most active in micelle formation was designed. The analysis of the influence of various structural factors on the value of the critical micelle concentration was supported by atomic charge studies. The obtained model will be used to design new star-shaped trimeric surfactants that are more active in formation of micelle as well as to predict their critical micelle concentration.

## Introduction

Oligomeric surfactants with multiple hydrophobic and hydrophilic groups are a new class of surfactants which become attractive in recent years. Dimeric surfactant, also called gemini surfactants, are simplest oligomeric surfactants which contain two hydrophobic and two hydrophilic groups connected by the spacer group. Trimeric surfactants are natural extension of the gemini surfactants. Their structure represent intermediate structure between dimeric surfactants and higher oligomeric ones. The first report on trimeric surfactants was by Raoul Zana et al.^1–3^. Generally, the oligomeric surfactants are constructed by two or more hydrophobic tails and polar head groups linked by the spacer groups. The spacer groups of trimeric and higher oligomerization degree surfactants can be linear, ring-like or star-shaped therefore the oligomeric surfactants are categorized into linear, ring-like and star-shaped, and their aggregation behavior strongly depends on their topological structures^4^. The star-shaped trimeric surfactants exhibit more unique self-aggregation behavior in aqueous solution compared to their linear analogues^4^.

Generally, the trimeric surfactants show an excellent surface-active and self-aggregation properties in aqueous solutions. The experimental studies^5–9^ and also the theoretical considerations^10^ show that the critical micelle concentration decreases with the increasing the degree of oligomerization. Thus, the critical micelle concentration of trimeric surfactants is much lower than those of the corresponding monomeric and dimeric analogues. Also, the cationic trimeric compounds exhibit strong antimicrobial activity. They are active against board range of microorganism such as bacteria and fungi^11,12^ and probably can be used against some viruses.

The main aim of this work is designing the structure of cationic star-shaped trimeric surfactants most active in micelle formation otherwise to study the effect of the structure modifications on the critical micelle concentration value. This work is a continuation of studies on structure–property relationship (QSPR) of cationic surfactants^13–15^. The subject of the previous studies was the critical micelle concentration (*cmc*) of cationic monomeric^13^ and cationic gemini^14,15^ surfactants. In the papers^13–15^, the relationship of the critical micelle concentration and the structure of cationic surfactants was investigated using the molecular connectivity indices only. In the present work these topological indices were also used to study the influence of the chains structure on the value of critical micelle concentration of star-shaped cationic trimeric surfactants. As was suggested in paper^4^ the topological structure of the oligomeric surfactants strongly affect the aggregation behavior of these compounds. Therefore, the topological descriptors like the molecular connectivity indices^16^ can be very good representation of the oligomeric surfactant structure in studies of self-aggregation properties, and in particular of the critical micelle concentration, as was shown in the previous papers concerning the gemini surfactants^14,15^.

The critical micelle concentration depends not only on geometrical structure but also on a number of other parameters, among them a kind of counterion. Therefore, in order to minimize the influence of factors other than geometrical on *cmc* value, only the star-shaped cationic surfactants with bromides as counterions were taken into account. Although, the obtained QSPR model has been derived for compounds with fixed counterions, it can be used to design the structure of cationic trimeric star-shaped surfactants with any kind of counterion because the impact of modifying the geometrical structure on changes in the *cmc* value, its decrease or increase, should be the same.

The semi-empirical calculations of the atomic charges were also performed to investigate the effect of branches and heteroatoms contained in the spacer group on the critical micelle concentration of studied trimeric surfactants.

## Results and discussion

All investigated cationic trimeric surfactants are star-shaped type surfactants. The geometrical structures of investigated compounds are significantly different. The entire data set includes thirteen training set compounds (compounds **1**–**13**) and five test compounds (compounds **T1**–**T5**). The structures of the molecules taken from literature along with the logarithms of the literature *cmc* values are shown in Methods section.

Molecular connectivity indices were calculated basing on the graphic structural formula of the molecules **1**–**13** using the expressions contained in Methods section (Eqs. 3–5). The values of five molecular connectivity indices from zero to fourth order and five valence molecular connectivity indices from zero to fourth order of training compounds are given in Table 1.

**Table 1:** Values of molecular connectivity indices.

No	${}^{0}\chi$	${}^{1}\chi$	${}^{2}\chi$	${}^{3}\chi_{c}$	${}^{4}\chi_{pc}$	${}^{0}\chi^{\nu }$	${}^{1}\chi^{\nu }$	${}^{2}\chi^{\nu }$	${}^{3}\chi_{c}^{\nu }$	${}^{4}\chi_{pc}^{\nu }$
1	36.53319	23.46739	19.70997	3.82544	4.05433	36.2447	22.65065	18.47576	3.39712	3.57442
2	40.77583	26.46739	21.83129	3.82544	4.05433	40.48734	25.65065	20.59708	3.39712	3.57442
3	45.01847	29.46739	23.95261	3.82544	4.05433	44.72998	28.65065	22.71840	3.39712	3.57442
4	43.38657	27.64893	23.75389	4.69147	4.76746	40.19419	24.30208	19.38003	3.57390	3.30413
5	51.87185	33.64893	27.99653	4.69147	4.76746	48.67947	30.30208	23.62267	3.57390	3.30413
6	56.11449	36.64893	30.11785	4.69147	4.76746	52.92211	33.30208	25.74399	3.57390	3.30413
7	44.54127	28.59842	25.07538	5.09972	5.98618	40.23638	23.89611	19.00814	3.65964	3.82895
8	53.02655	34.59842	29.31802	5.09972	5.98618	48.72167	29.89611	23.25078	3.65964	3.82895
9	44.88657	28.89157	27.21624	6.81279	7.88938	41.82432	26.00647	22.85695	5.06880	5.50002
10	49.12921	31.89157	29.33757	6.81279	7.88938	46.06697	29.00647	24.97828	5.06880	5.50002
11	32.49730	22.69453	17.90271	1.42887	2.87217	30.41853	19.56576	13.81274	0.79057	1.43061
12	33.65200	23.64402	19.31693	1.83712	3.42036	31.08205	19.90978	14.43237	0.98601	1.54138
13	50.17281	34.23886	27.51366	2.80888	5.40758	44.53661	28.39050	20.17867	1.22422	1.86717

Based on the values of connectivity indices (Table 1) and the logarithms of literature *cmc* values of training compounds **1**–**13**, using the polynomial regression analysis and stepwise method, the two-variable equation (Eq. 1), expressing the relation between logarithm of *cmc* and the molecular connectivity indices, have been obtained:

1 $$ Log_{10} cmc = - (1.10878 \mp 0.18486) + (0.00428 \pm 0.00058)\, \cdot \,({}^{2}\chi )^{2} - (0.00709 \mp 0.00055)\, \cdot \,({}^{1}\chi^{\nu } )^{2} $$

The statistical characteristics of the equation variables are given in Table 2.

**Table 2:** Statistical characteristics of variables included in Eq. (1).

Variable	Coefficient	Standard error	*t*-value	*p*-value
Constant	− 1.10878	0.18486	− 5.9979	0.00013
$({}^{2}\chi )^{2}$	0.00428	0.00058	7.3276	0.00003
$({}^{1}\chi^{\nu } )^{2}$	− 0.00709	0.00055	− 12.9841	0.00000

The obtained model (Eq. 1) contains first-order valence molecular connectivity index ${}^{1}\chi^{\nu }$ and second-order molecular connectivity index ${}^{2}\chi$. The first-order valence molecular connectivity index ${}^{1}\chi^{\nu }$ is path-type index and it represents one-bonds fragments in a molecule. The values of this index depend on the isomers of the compound^16^ and decrease with increasing in branching. The molecular valence connectivity index ${}^{1}\chi^{\nu }$ is a valence connectivity index thus differentiates heteroatoms and multiple bonds. The second-order ${}^{2}\chi$ molecular connectivity index appearing in the model (Eq. 1) represents two-bonds fragments in the molecule. The values of this index also depend on the isomers of the compound^16^, and in this case the values increase with increasing in branching. Also, the values of ${}^{2}\chi$ and ${}^{1}\chi^{\nu }$ indices increase with the increase in the number of atoms in the molecule by extending the hydrocarbon chains or adding atoms to the chains through branching.

From Table 1 it can be concluded that the values of ${}^{2}\chi$ index are smaller compared to the values of ${}^{1}\chi^{\nu }$ index. The obtained model (Eq. 1) contains the $({}^{1}\chi^{\nu } )^{2}$ variable with a negative coefficient and the $({}^{2}\chi )^{2}$ variable with a positive one, and the absolute value of these coefficients are 0.00709 and 0.00428, respectively. Therefore, the analysis of equation (Eq. 1) allows us to conclude that as the ${}^{1}\chi^{\nu }$ index increases, the *cmc* decreases.

**Figure 1:**
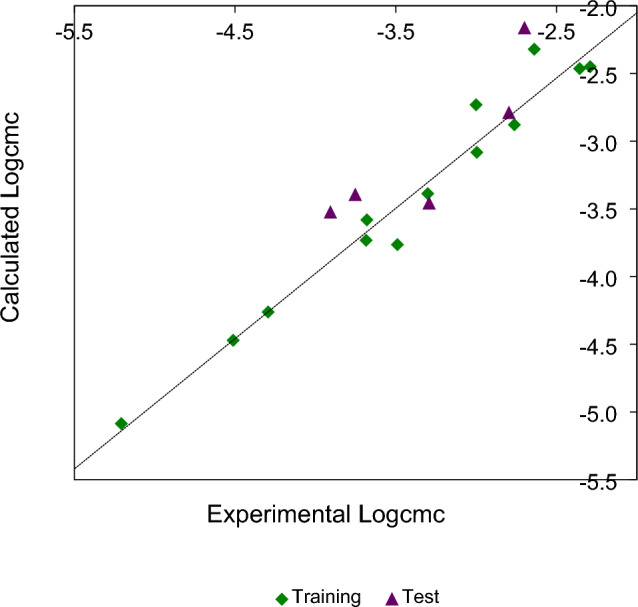
Scatter plot of the calculated $Log_{10} cmc$ versus the experimental $Log_{10} cmc$ for compounds of training set (*r* = 0.981, *F* = 280.480, *s* = 0.173, *r*^*2*^_*adj*_ = 0.959, $Q_{LOO}^{2}$ = 0.925) (rhombus) and test set ($R_{pred}^{2}$ = 0.531) (triangle).

The graphical comparison of the calculated $Log_{10} cmc$ values using Eq. (1) and the experimental $Log_{10} cmc$ values is shown in Fig. 1.

The $Log_{10} cmc$ values calculated using Eq. (1) and the experimental $Log_{10} cmc$ values for studied trimeric surfactants, along with the values of residuals, are contained in Table 3.

**Table 3:** Calculated and literature values $Log_{10} cmc$ of compounds from training and test set.

Compound	Experimental $Log_{10} cmc$	Calculated $Log_{10} cmc$	Residual
1	− 2.99568^17^	− 3.08207	0.08639
2	− 3.68403^17^	− 3.73189	0.04786
3	− 4.51145^17^	− 4.47077	− 0.04068
4	− 2.76195^18^	− 2.87906	0.11711
5	− 4.29414^18^	− 4.26129	− 0.03285
6	− 5.20761^18^	− 5.08599	− 0.12162
7	− 2.35556^19^	− 2.46403	0.10847
8	− 3.48945^19^	− 3.76371	0.27426
9	− 3.00000^20^	− 2.73117	− 0.26883
10	− 3.30103^20^	− 3.38734	0.08631
11	− 2.28988^21^	− 2.44998	0.16010
12	− 2.63827^21^	− 2.32084	− 0.31743
13	− 3.67985^22^	− 3.58075	− 0.09910
T1	− 2.79588^23^	− 2.79056	− 0.00532
T2	− 3.75203^23^	− 3.39585	− 0.35618
T3	− 3.29243^24^	− 3.45880	0.16637
T4	− 3.90658^18^	− 3.52565	0.38093
T5	− 2.69897^20^	− 2.16405	0.53492

**Figure 2:**
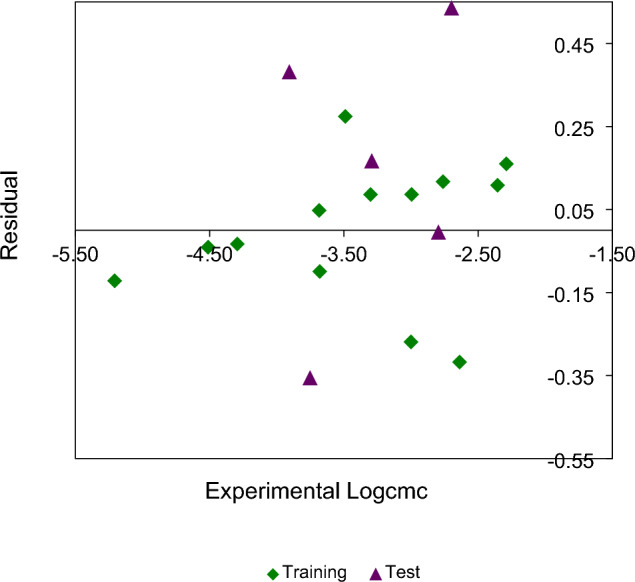
Plot of residuals versus the experimental $Log_{10} cmc$ values for training set (rhombus) and test compounds (triangle).

The plot of residuals versus the experimental values of $Log_{10} cmc$ is shown in Fig. 2.

As can be seen from Table 3 and Figs. 1–2, the calculated values of $Log_{10} cmc$ using Eq. (1) are very close to the from literature ones.

As shown in Table 6 given in Methods section, the Eq. (1) has been obtained based on molecules with a different topological structure. These compounds differ in the length of the hydrophobic chains as well as the structure of spacer groups. Therefore, it is worth analyzing, based on the obtained equation, the influence of hydrophobic tails and spacer group on the values of logarithm of *cmc*.

### Hydrophobic tails effect

**Figure 3:**
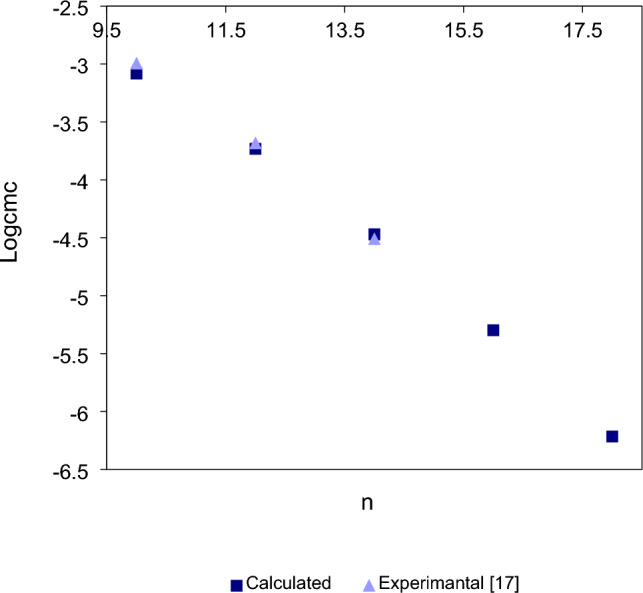
Calculated (square) and experimental^17^ (triangle) $Log_{10} cmc$ values of star-shaped trimeric compounds **1**–**3** versus the number of alkyl chain carbon atoms (n).

**Figure 4:**
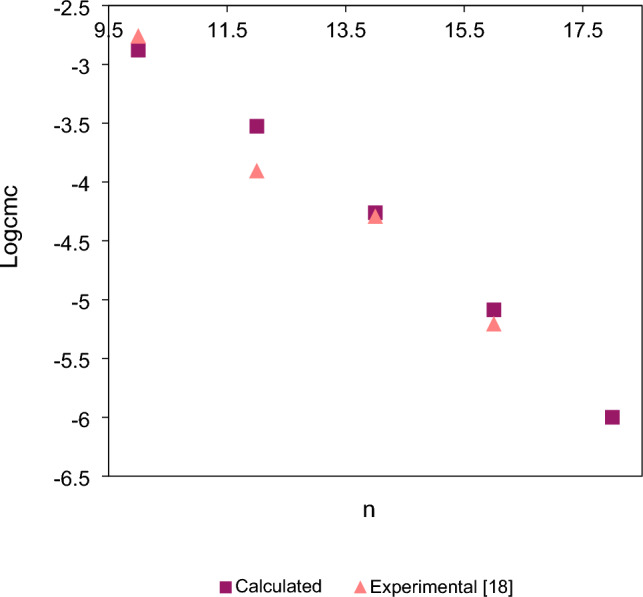
Calculated (square) and experimental^18^ (triangle) $Log_{10} cmc$ values of star-shaped trimeric compounds **5**–**6** and **T4** versus the number of alkyl chain carbon atoms (n).

The experimental *cmc* values and thus $Log_{10} cmc$ of star-shaped cationic trimeric surfactants decrease when the tails are lengthened between ten and fourteen (compounds **1**–**3**) or ten and sixteen (compounds **4**–**6** and **T4**) carbon atoms. The corresponding plots of the calculated, using Eq. (1), and the experimental $Log_{10} cmc$ versus the alkyl chain carbon number of compounds **1**–**3** and compounds **4**–**6** and **T4** are shown in Figs. 3–4.

As shown in Figs. 3–4, the $Log_{10} cmc$ values calculated using Eq. (1) also decrease with tails lengthening from ten to eighteen (Figs. 3–4) carbon atoms, and as shown in Figs. 3–4, with this range the dependence of both the calculated and also experimental logarithm values of *cmc* on the number of carbon atoms in the alkyl chains is linear. As can be seen in Fig. 3–4, the calculated $Log_{10} cmc$ values are very close to experimental values. For experimental $Log_{10} cmc$ values of compounds in Fig. 4 there is a little deviation from linearity for the twelfth carbon atoms chain, but this deviation is probably within the margin of experimental error. Thus, it can be concluded that in the range between the ten and the eighteen carbon atoms the dependence of $Log_{10} cmc$ on the number of alkyl chain carbon atoms (n) of star-shaped cationic trimeric surfactants can be described by linear function.

However, in the case of longer chains, above eighteen carbon atoms, the dependence of the $Log_{10} cmc$ values calculated using Eq. (1) on the number of carbon atoms will be non-linear due to the non-linear nature of this equation. The non-linear dependence of logarithm of *cmc* on the chains length has been observed for various cationic gemini surfactants of different nature and flexibility spacer groups or tails^25–27^. Also, the non-linear relationship between $Log_{10} cmc$ and the number of tail carbon atoms for cationic gemini surfactants was obtained basing on theoretical model^28^.

### Spacer group effect

The structure and nature of the spacer group play an important role in micelle formation of oligomeric surfactants. The studies on gemini surfactants^29–33^ show that such features of the spacer group as flexibility and hydrophobicity have significant impact on the aggregation behavior in aqueous solution of these compounds. Therefore, the effect of such factors which influence the flexibility and hydrophobicity as spacer’s length, branching and heteroatoms on the *cmc* of star-shaped trimeric surfactants have been investigated using Eq. (1) (Table 4). In Table 4 the star-shaped type trimeric surfactants with different spacers groups structures and corresponding $Log_{10} cmc$ values calculated using Eq. (1) are shown.

**Table 4:** Calculated $Log_{10} cmc$ values of star-shaped trimeric surfactants with different spacer group (**S1**-**S10**).


Compound	X	Y	Calculated $Log_{10} cmc$
S1		–CH_2_–	− 4.80429
S2		–CH_2_–	− 4.43451
S3	CH	–CH_2_–	− 4.09246
S4	N	–CH_2_–	− 4.01400
S5	N	–NH–	− 3.75740
S6	N	–O–	− 3.61353
S7	N	–CH_2_–CH_2_–	− 4.47315

S8	N	–CH_3_	− 3.78889
S9	N	–NH_2_	− 3.51421
S10	N	–OH	− 3.43116

The inspection of data contained in Table 4 shows that the greatest value of critical micelle concentration is for compound **S6** and the lowest for compound **S1**.

In the case of compounds with different central groups in the spacer group (compounds **S1**–**S4**), the lowest *cmc* value is for **S1** compound with the cyclohexane-based spacer group, and the highest for compound **S4** with the central nitrogen atom in the spacer group. This lowest *cmc* value of compound **S1** may be due to the fact that the spacer is more hydrophobic which may cause its incorporation into the interior of the formed micelle. Also, as shown in Table 4, the *cmc* value of compound **S2** is lower than *cmc* value of compound **S4**. Similar results was obtained by Nacham et al.^21^. The authors of paper^21^ have studied among others the ionic liquid-based star-shaped trimeric surfactants containing triethylamine and triethylbenzene spacer groups. As was shown in^21^ the *cmc* value of IL-based trimeric surfactant containing triethylbenzene spacer groups is lower than the *cmc* of that containing triethylamine spacer group with identical alkyl tails and head groups. The authors of this paper also suggest that this difference of *cmc* values may be due to the higher hydrophobicity imparted by the benzyl core in investigated IL-trimeric surfactant. Table 4 also shows that *cmc* value of compound **S1** is lower compared to the *cmc* value of compound **S2**. This difference may result from the fact that substituted cyclohexane ring is more flexible compared to the benzene one, and thus the cyclohexane-based spacer group easily incorporates into the interior of the formed micelle.

Among the compounds having nitrogen as the central group and the same number of non-hydrogen atoms in the spacer group (compounds **S4**–**S6** and **S8**–**S10**), the lowest critical micelle concentration is for compound **S4** and the greatest for compound **S10**. In the case of the compounds with straight spacer chains (compounds **S4**–**S6**), the *cmc* value increases in the order: –CH_2_– < –NH– < –O–. The similar order is for compounds with branched spacer group (compounds **S8**–**S10**), the order of increasing *cmc* value is: –CH_3_ < –NH_2_ < –OH. In addition, the comparison of the critical micelle concentration values of compounds with straight chains of star-shaped spacers (compounds **S4**–**S6**) with the corresponding compounds having branched spacer group (compounds **S8**–**S10**) shows that the critical micelle concentration values of compounds having the branched spacer groups are greater compared to the *cmc* values of the corresponding compounds with straight spacer’s chains.

In summary, Table 4 shows that in the case of star-shaped spacers having the same number of non-hydrogen atoms, the branches and the heteroatoms cause the increase the critical micelle concentration value.

The effect of spacer’s branches and heteroatoms on *cmc* value of compounds presented in Table 4 (compounds **S4**–**S6** and **S8**–**S10**) was also analyzed using the atomic charges. The total charges of different spacer’s functional groups found in the **Y** position of compounds **S4**–**S6** and **S8**–**S10** are presented in Table 5.

**Table 5:** Total charges of the different functional groups found in the Y position (see Table 4).

Straight spacer (S4–S6)				Branched spacer (S8–S10)			
Group	Charge			Group	Charge		
	Y_1_	Y_2_	Y_3_		Y_1_	Y_2_	Y_3_
–CH_2_–	0.0057	0.0179	0.0435	–CH_3_	0.0599	0.0514	0.0550
–NH–	− 0.211	− 0.1377	− 0.1263	–NH_2_	− 0.0065	− 0.0293	− 0.030
–O–	− 0.3271	− 0.2867	− 0.2987	–OH	− 0.099	− 0.0961	− 0.1037

The AM1 semi-empirical calculations (Table 5) show that the atoms of high electronegativity such oxygen and nitrogen introduce a great negative charge to the spacer group, greater than the carbon atom thus the total charges of functional groups containing heteroatoms are negative. Moreover, the total charges of the various functional groups shown in Table 5 show that for compounds both straight and branched spacer group the total charge changes from positive for functional group with carbon atom to negative for functional groups with nitrogen or oxygen. Additionally, the negative charge is greater in the case of a group with an oxygen atom than with a nitrogen atom. Comparing these results with the data from Table 4, it can be concluded that the *cmc* values increase with the increase in the negative charge of the functional group.

The data in Table 5 also show that the total (negative) charge of the functional group, in the branched spacer chain compared to that of the corresponding functional group in the straight chain, is lower for branched spacer when the non-hydrogen atom in the functional group in Y position is oxygen or nitrogen, and greater (positive) charge also for branched spacer group when the non-hydrogen atom in the functional group in Y position is carbon atom. Comparing these results with the data from Table 4, it can be seen that when the non-hydrogen atom in the functional group is carbon atom, the results from Table 5 agree very well with those in Table 4 discussed above, i.e. the greater the positive charge, the greater the critical micelle concentration. In the case of functional groups containing heteroatoms (Table 4), it can be seen that the *cmc* value is greater for compounds with branched spacer group for which the negative charge of the functional group is lower than the negative charge of analogous functional group in straight spacer chain (Table 5).

The obtained model also shows that the *cmc* value of star-shaped trimeric surfactants decreases with the increase in the chains length of the spacer group (compounds **S4** and **S7**). This is due to the fact that changes in the length of hydrocarbon tails have a significant impact on *cmc* values, and thus on obtained model. However, the experimental data^34^ shows the opposite conclusion. As shown in^34^, the *cmc* value of 3C_12_tris-s-Q cationic trimeric surfactants is greater for s = 6 than for s = 3. This suggests that the *cmc* increases with increasing spacer chains length (s) from three to about six methylene groups. A similar effect of spacer chains length is observed for dimeric surfactants^29,30^. The *cmc* values of 12–s–12 gemini surfactants increase with increasing number of spacer carbon atoms, up to four or five methylene groups, and then decrease with further elongation of the spacer chain^29,30^. Therefore, probably the increase in *cmc* value of trimeric surfactants with spacer chains length may have maximum at about six carbon atoms and then, as for gemini surfactants, the *cmc* may decrease with further chains length elongation. To the best of the author's knowledge, spacer chains lengthening above six methylene groups for star-shaped trimeric surfactants has not been studied. Therefore, the obtained model should be used probably for compounds with more than six carbon atoms in each chain of the trimeric surfactant spacer group when examining the effect of the spacer chains elongation on the *cmc* value.

Finally, if we examine only the changes in *cmc* value due to changes in the geometrical structure, the obtained equation (Eq. 1), although derived for bromide compounds, can also be used for compounds with another counterion, because the changes in *cmc*, its increase or decrease, should be the same. In other words, when examining changes in the *cmc* of bromide star-shaped trimeric surfactants, we can expect that in the case of chloride analogous compounds will be similar. For example, for chloride compounds 1,1,1-Tris[2-hydroxy-3-(dodecyldimethylammonio)-propoxymethyl]ethane Trichloride^35^ and the so-called III-12-4 trimeric surfactant^36^, for which the experimental *cmc* values are 0.223 (mM) and 0.15 (mM), respectively, the calculated values using Eq. (1) are 0.198 (mM) and 0.187 (mM), respectively. So, the calculated *cmc* value decreases as the experiment shows.

## Methods

### Experimental data

All investigated molecules are trimeric quaternary ammonium bromide surfactants. The structures of all considered compounds along with the logarithms of the literature *cmc* values^17–24^ are shown in Table 6.

**Table 6:** Molecular structures and logarithms of experimental *cmc* values of training (**1**–**13**) and test (**T1**–**T5**) compounds.

No	Molecular structure	n	$Log_{10} cmc$	Ref
1 2 3		10 12 14	− 2.99568 − 3.68403 − 4.51145	^17^
4 5 6		10 14 16	− 2.76195 − 4.29414 − 5.20761	^18^
7 8		8 12	− 2.35556 − 3.48945	^19^
9 10		10 12	− 3.00000 − 3.30103	^20^
11		8	− 2.28988	^21^
12		8	− 2.63827	^21^
13		12	− 3.67985	^22^
T1 T2		10 12	− 2.79588 − 3.75203	^23^
T3		12	− 3.29243	^24^
T4		12	− 3.90658	^18^
T5		8	− 2.69897	^20^

The literature values of critical micelle concentration were given in (mM)^17–24^. The *cmc* values expressed in molar units (M) have been converted to logarithms of *cmc* (Table 6).

Most values of *cmc* were measured at 25 °C in aqueous solution. The *cmc* of compound **T3** was measured at 22 oC.

### Molecular connectivity indices

Molecular connectivity indices, some of the topological descriptors to characterize molecules in structure–property and structure–activity studies, are calculated from the molecular graph. The molecular graph is a graphical representation of the structural formula of the chemical compound, in which vertices represents atoms and edges symbolize covalence bonds.

The first connectivity index was proposed by Randić^37^ and was defined as:

2 $$ \chi = \sum\limits_{k} {\left( {\delta_{i} \delta_{j} } \right)}_{k}^{ - 0.5} $$

where $\delta_{i}$ is a connectivity degree i.e. the number of non-hydrogen atoms to which the *i*-th non-hydrogen atom is bonded. The Kier and Hall molecular connectivity indices^16^ are generalizations of Randić’s connectivity index and the *m*-th order Kier-Hall molecular connectivity index is defined as^16^:

3 $$ {}^{m}\chi_{k} = \sum\limits_{j = 1}^{{n_{m} }} {\prod\limits_{i = 1}^{m + 1} {\left( {\delta_{i} } \right)_{j}^{ - 0.5} } } $$

where $\delta_{i}$ is a connectivity degree, *m* is the order of the connectivity index, *k* denotes type of the fragment of the molecule for example: path (p), cluster (c) and path-cluster (pc), *n*_*m*_ is the number of fragments of type *k* and order *m*.

The *m*-th order valence molecular connectivity index is defined^16^:

4 $$ {}^{m}\chi_{k}^{\nu } = \sum\limits_{j = 1}^{{n_{m} }} {\prod\limits_{i = 1}^{m + 1} {\left( {\delta_{i}^{\nu } } \right)_{j}^{ - 0.5} } } $$

where the valence connectivity degree $\delta_{i}^{\nu }$ is defined as:

5 $$ \delta_{i}^{\nu } = \frac{{Z_{i}^{\nu } - h_{i} }}{{Z_{i} - Z_{i}^{\nu } - 1}} $$

where $Z_{i}^{\nu }$ is the number of valence electrons in the *i*-th atom, *h*_*i*_ is the number of hydrogen atoms connected to the *i*-th atom and $Z_{i}$ is the number of all electrons in the *i*-th atom.

The values of the molecular and valence molecular connectivity indices of training compounds are contained in Table 1.

### Atomic charges

Atomic charges were calculated using the semi-empirical molecular orbital package MOPAC 7 included in the VEGA program^38,39^, employing the semi-empirical AM1 method. All calculated atomic charges are expressed in atomic units (a.u.).

### Statistics

Each formula expressing the relationship between the $Log_{10} cmc$ and molecular connectivity indices was generated using the least-squares method and the final equation was obtained using the stepwise method. Pearson correlation coefficient (*r*), the adjusted coefficient of determination (*r*^*2*^_*adj*_), the standard deviation of the fit (*s*) and the Fisher ratio (*F*) were used to select the best model. The model obtained was selected according to following principles: highest correlation coefficient, adjusted coefficient of determination and Fisher value, the lowest standard deviation of the fit and also the smallest possible number of significant descriptors in the model. For good quality QSPR model the positive value of correlation coefficient (*r*) should be closer to 1. High values of *F*-test indicate that the model is statistically significant. The number of variables in the model should not exceed the number of compounds divided by five^40^. The statistical characteristics of the equation variables includes standard error, t-value and p-value. High absolute Student *t* value of the variable expresses that the coefficient of the variable is significantly larger than the standard error. Variable with *p* value below 0.05 is considered statistically significant^41^.

The leave-one-out cross-validated correlation coefficient ($Q_{LOO}^{2}$) and predictive correlation coefficient ($R_{pred}^{2}$) were also used to indicate the internal and external validation of derived model. The model is considered to be excellent, if $Q_{LOO}^{2}$ is equal or more than 0.9^42^. For acceptable QSPR model, the values of $Q_{LOO}^{2}$ and $R_{pred}^{2}$ should be more than 0.5^40,43^.

The statistical calculations were made using the program STATISTICA 12^44^.

## Conclusions

The aim of the presented work was to find a simple equation expressing the dependence of the critical micelle concentration of cationic star-shaped trimeric surfactants on their geometrical structures represented by topological indices, that will allow for examining the impact of structure modifications on the *cmc* values and thus it can be helpful in the design of novel star-shaped cationic trimeric surfactants. Employing the polynomial regression analysis, the equation with two molecular connectivity indices was obtained. Using the obtained model, the influence of hydrophobic tails length and structure of star-shaped spacer group on the critical micelle concentration was examined.

The analysis of the influence of tails length in the range between ten and eighteen carbon atoms confirmed the experimental results and the Klevens equation^45^ which expresses a linear dependence of logarithm of *cmc* on the number of carbon atoms in the alkyl chains.

The analysis of star-shaped spacers with the same number of non-hydrogen atoms allows us to conclude that heteroatoms cause the increase the *cmc*. These results were confirmed by atomic charge analysis. Functional groups containing highly electronegativity atoms, such as oxygen or nitrogen, introduce into the spacer group the negative charges, which cause an increase in the critical micelle concentration. The spacer group analysis also reveals that the branches cause the increase the critical micelle concentration too.

## Data Availability

All data generated or analysed during study are included in this published article. The experimental data were taken from literature.
